# Mitochondrial DNA in Human Diversity and Health: From the Golden Age to the Omics Era

**DOI:** 10.3390/genes14081534

**Published:** 2023-07-27

**Authors:** Candela L. Hernández

**Affiliations:** Department of Biodiversity, Ecology and Evolution, Faculty of Biological Sciences, Complutense University of Madrid, 28040 Madrid, Spain; clhernan@bio.ucm.es

**Keywords:** mitogenome, phylogeny, phylogeography, haplogroup, human population genetics, forensics, aDNA, diseases, longevity, aging

## Abstract

Mitochondrial DNA (mtDNA) is a small fraction of our hereditary material. However, this molecule has had an overwhelming presence in scientific research for decades until the arrival of high-throughput studies. Several appealing properties justify the application of mtDNA to understand how human populations are—from a genetic perspective—and how individuals exhibit phenotypes of biomedical importance. Here, I review the basics of mitochondrial studies with a focus on the dawn of the field, analysis methods and the connection between two sides of mitochondrial genetics: anthropological and biomedical. The particularities of mtDNA, with respect to inheritance pattern, evolutionary rate and dependence on the nuclear genome, explain the challenges of associating mtDNA composition and diseases. Finally, I consider the relevance of this single locus in the context of omics research. The present work may serve as a tribute to a tool that has provided important insights into the past and present of humankind.

## 1. Introduction

Mitochondria are cellular components brought recurrently to the forefront of scientific research in evolutionary biology, biological anthropology, molecular genetics, biochemistry, biomedicine and gerontology. A fascinating tale explains the presence of this organelle within (human) cells, as the result of an endosymbiotic relationship between alphaproteobacteria and host ancestral cells [1]. Mitochondria harbor genetic material, the remnant of the original bacterial genomes that co-evolved with the nuclear genome of proto-eukaryotic cells. Mitochondrial DNA (mtDNA) plays a key role in many physiological processes, since mitochondria is involved in a mosaic of essential biological functions including the generation of cellular energy [2]. Therefore, variants in mtDNA have likely important consequences on human biology [3] and we can study their effects through multiple facets.

Changes in the mtDNA sequence have been related to environmental adaptation in recent human evolution; hence, selective forces must be taken into account when analyzing mitochondrial inter-population diversity [4,5,6]. 

Furthermore, to deepen the understanding of our own past, mtDNA was the tool by which a scientific consensus emerged regarding the common African genetic origin of all present-day human populations around 200 thousand years ago (kya) [7]. In their groundbreaking study, Rebeca L. Cann and co-authors traced phylogenetic relationships of continental areas and, interestingly, revealed a higher genetic variability in sub-Saharan African populations. Afterwards, the mitochondrial genome—together with the non-recombining region of the Y-chromosome, NRY—has lead human population genetic studies for roughly 25 years. As it is exposed along this survey, mtDNA is undoubtedly the molecular target that has intensely shaped the current knowledge of contemporary human diversity on a global scale.

Mutational events of the mtDNA sequence are a major cause of inherited human disease [8]. Mitochondrial diseases are the only genetic disorders in humans where two different genomes are involved: mtDNA and nuclear DNA (nDNA) [9]. On the other hand, since the definition of the *mitochondrial free radical theory of aging* (MFRTA) [10], mtDNA has been also considered as a main player for understanding human aging and longevity.

The present review explores the productive field of human mitochondrial genetics throughout 40 years of active research. Concretely, this work aims to combine two main perspectives of mito-studies: the population view (comprising concepts such as phylogenetics, inherited variation and germinal line) and mitochondrial diseases (where somatic variation should be strongly considered). Finally, the following questions are addressed: is mtDNA still a relevant tool in the late post-genomic age? How could this molecule face the advent of new sequencing technologies or, more recently, multi-omics approaches to analyze human diversity and health?

## 2. The Mitochondrial Genome: Living in a Singular Intracellular Context

The human mitogenome is a circular double-stranded molecule spanning 16,569 base pairs (bp). Most of mtDNA (~90%) is composed of coding regions. The remaining fragment is known as the displacement loop (*D-loop*) or the control region, as it is involved in the regulation of mitogenome replication and transcription. Since the 1100 bp comprising the *D-loop* is highly polymorphic, this fragment is also known as the *hypervariable region* (HVR) [11].

Although the vast majority of the ~1000 mitochondrial proteins are coded in the nucleus, 13 of them are coded in the mitochondrial genome (*mitogenome*), as they are essential for both the respiratory chain and oxidative phosphorylation (OXPHOS). The remaining 24 genes contained in the mtDNA molecule are associated with the translational machinery of the molecule [12,13].

It can be stated that mtDNA lives in an ‘apparent paradox’. Given the relevant bioenergetic role of mitochondria and its involvement in several cellular processes such as signaling, homeostasis, cell growth or inflammation, mtDNA genes should be highly conserved. However, mtDNA has a very high sequence evolutionary rate [3]. This is just one example of the high complexity of mitochondria as a biological entity (further discussed in Section 4).

Because of the specific location within the cell, several traits distinguish mtDNA from nDNA. These interconnected features justify the use of the mitochondrial genome in several fields of biology—human population genetics, forensic sciences and paleogenomics—and biomedicine. 

### 2.1. High Evolutionary Rate

The human mitochondrial genome evolves at a rapid rate. First attempts to calibrate the mitochondrial molecular clock in primates estimated 2 × 10^−8^ substitutions per bp per year, which is 5- to 10-fold higher than the nDNA rate [14]. Since this parameter is the key to setting coalescence ages and time boundaries of major events in the history of modern humans, many efforts have been devoted to unraveling the mtDNA change rate [4,6,15,16,17,18,19,20]. In the last few years, some studies have addressed this issue by combining modern mitogenomes with the growing evidence of ancient DNA (aDNA) data, using sequences with reliable radiocarbon dates associated [21,22,23,24]. Some points raised by these studies can be summarized as follows: (*i*) non-homogeneous rates are observed along the molecule—control vs. coding regions; (*ii*) a higher proportion of synonymous mutations are detected in ancient branches from mtDNA phylogeny when compared to younger ones—the result of a purifying selection acting over time or the recent relaxation of selective constraints; (*iii*) globally, the evolutionary rate of recent mitochondrial sequences is accelerated, demonstrating a time dependency effect, and (*iv*) intra-lineage rate variation, demonstrated in some haplogroups, is possibly due to different population demographic histories. Therefore, human mitogenomes have evolved dynamically following intertwined processes. 

The inherited mitochondrial change linked to mutations occurred in the germline—with evolutionary consequences—should be complemented with the other source of variation: the somatic one. Mutations accumulate in the mtDNA sequence throughout the human lifetime, as we will explore below, in the frame of the oxidative stress due to mitochondrial physiological processes [13,25,26,27]. 

### 2.2. High Copy Number per Cell

Each human cell can contain hundreds or thousands of copies of mtDNA. The polyploidy nature of this genome is the basis of a main characteristic of the molecule: the possible presence of a mixture of mtDNA types. All copies can be identical (*homoplasmy*) or different (*heteroplasmy*) [28]. The presence and proportion of the mutated genome with respect to wild-type mtDNA is substantial when dealing with the clinical expression of mitochondrial diseases. It is known that the level of heteroplasmy can be modified sharply during germline transmission, due to the mtDNA genetic bottleneck (only a small proportion of the total maternal mitogenomes are passed on to offspring) [29].

The presence of multiple copies per cell justified that mtDNA is a primary molecular resource in forensics and aDNA studies, which are fields limited by the quality and quantity of the target DNA [30]. MtDNA was indeed present at the foundation of paleogenetics [31] and for solving relevant cases of human identification [32].

### 2.3. Maternal Inheritance

The uniparental inheritance is one of the greatest assets of mtDNA that, in combination with the absence of recombination, allows direct reconstructions of maternal lineages back in time [33]. This situation mirrors that which occurred for the other genomic haploid system, the NRY, which represents the paternal history of humankind. 

The particular maternal transmission of human mtDNA was firstly described in 1980 [34]. Evidences of specific mechanisms that mark sperm mitochondria in the oocyte for destruction have been found; this phenomenon could have an evolutionary explanation, preventing paternal and maternal genome competition [35]. Nevertheless, the strict female transmission to offspring has been questioned. Schwartz and Vissing [36] reported the case of a male with a mitochondrial myopathy caused by a paternally inherited mutation in gene *MT-ND2*. More recently, other authors described the biparental inheritance of mtDNA haplotypes in three multi-generational pedigrees [37]. There is an interesting debate on whether these cases reflected actual violations of mtDNA transmission. Methodological issues (contamination) or amplifications of nuclear-encoded mitochondrial sequences (NUMTs) have been suggested to explain the observed results [38,39].

### 2.4. Absence of Recombination

Other mitochondrial singularities—with respect to the nuclear genome—rely on its non-recombining nature. Although mitochondria contain the machinery needed for this process, recombination, both intergenomic (different mtDNA molecules) and reciprocal (same molecule) are rare phenomena in animals. Some punctual evidence has been found in somatic tissue [40], though without relevance for the offspring [41].

The mixture of several features, lack of recombination, uniparental transmission and the mitochondrial bottleneck that occurred during oogenesis, explains that the mtDNA molecule is severely affected by genetic drift. The effective number of alleles is approximately 1/4 for mtDNA relative to nDNA, meaning that mtDNA fixes new alleles faster than nDNA. Differences in that parameter, together with the total linkage of mtDNA, cause *selective sweeps* (the fixation of haplotypes as a consequence of the fitness advantage of one or more embedded variants) or *background selection* (the decrease in the effective population size due to the purifying of low-fitness variants), which could be considered as relevant phenomena [12].

## 3. Mitochondrial DNA Analysis: The Basis of Modern Human Population Genetics

In spite of representing a minor fraction of the human genetic material, mtDNA has been overrepresented in human population genetics from late 1980s until the beginning of the 2010s. New genetic variants appear more frequently than expected (due to the high mitochondrial evolutionary rate) and are indeed more prone to the effects of genetic drift (due to the absence of recombination and low effective size). In this context, the different mitochondrial lineages observed in specific populations evolve independently by sequential accumulations of mutations. This fact allows for a straightforward reconstruction of phylogenetic relationships among lineages within or across human populations. All the above-mentioned features explain that the human evolutionary past has been recurrently inspected by the use of mtDNA [42]. 

Next, fundamentals of human mitochondrial genetics from a population perspective are analyzed. 

### 3.1. References, Nomenclature and Consensuses

The publication of the human mtDNA sequence in 1981 [43] represented a key achievement for human genetics. The resulting *Cambridge Reference Sequence* (CRS) was re-sequenced afterwards, and the revised CRS (rCRS) was released in 1999 to correct some sequencing errors [44]. Since then, the rCRS was systematically used to refer to any new mutations. In 2012, an interesting initiative was launched to change the paradigm in mitochondrial studies [45]. Behar et al. described the *Reconstructed Sapiens Reference Sequence* (RSRS), built upon ~20,000 current mitogenomes and the information of the Neandertal mtDNA sequences published so far, that would be placed at the root of the human mitochondrial phylogeny. The main aim was to consider a hierarchical phylogenetic approach, where mtDNA variants were more consistently understood as ancestral or derived.

In spite of the great effort performed by the authors, this suggested switch did not totally permeate research. In the following years, several studies adopted the RSRS as a reference [46,47,48,49,50]; in others, however, both RSRS and rCRS coexisted [51,52,53,54] and, finally, a number of papers kept the original reference [21,55,56,57]. The replacement of the rCRS by the RSRS had some relevant dissent [58] with respect to the inherent notational problems raised when providing a new reference point for human mtDNA.

MtDNA variation, as an haploid marker, is studied in a phylogeographic perspective, that is, the connection between genealogy and geography [59]. The essential term in mitochondrial studies, *haplogroup*, was initially defined as a “group of related haplotypes” [60], and it has been equally represented by the words ‘lineage’, ‘clade’ or ‘cluster’. This core concept is more neatly defined by Pakendorf and Stoneking [33] as “*related groups of sequences that are defined by shared mutations and which tend to show regional specificity*”. Given this background, the detection of specific haplogroups in past- and present-day human populations testify in gene flow events and admixture processes with defined directions and demographic consequences [61].

**Figure 1:**
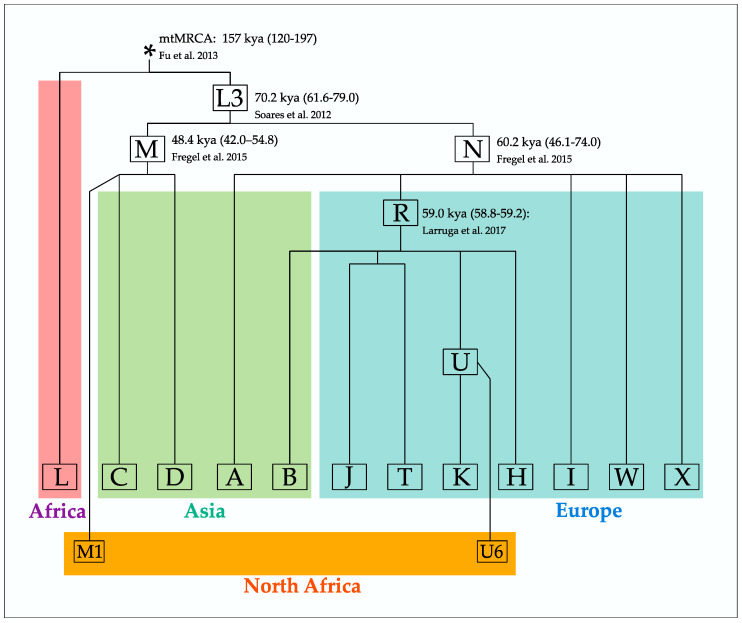
A simplified mtDNA phylogeny. Coalescence ages [point estimates (95% CI)] were retrieved from [21,56,66,67]. All estimates were based on mtDNA complete sequences, and the calibration rate is that proposed by Soares et al. [19], except for mtMRCA (*most recent common mitochondrial ancestor*).

The first mtDNA haplogroups (A, B, C, D) were described in Native American populations [62], and the same team defined lineages H, I, J and K soon after in American individuals with European ancestry [63]. With the characterization of haplogroups T, U, V, W, and X [64], the vast majority of the extra-African human mitochondrial diversity was described. All of the above-mentioned lineages are grouped in macro-haplogroup L3, the lineage that represents the out-of-Africa dispersion. Figure 1 presents the backbone of mtDNA phylogeny along with the evolutionary ages of some major branches. The clades nested within this tree are associated with their geographic origin and main distribution area. Two interesting sub-lineages, U6 and M1, are considered as markers of a North African native ancestry as a consequence of an Early Upper Paleolithic back migration from southwestern Asia to North Africa [65].

Early studies on the mtDNA diversity of human populations laid the framework for an emerging field that required specific cladistic rules. Richards et al. [68] provided some guidelines for the hierarchical structuring of haplogroups and sub-haplogroups. Haplogroups are denoted by capital letters and, as we are descending on the maternal genealogy, the branches are named after numbers and lower cap letters successively from the root to the tip branches (e.g., H, H1, H1a, H1a1…). Each sub-haplogroup is characterized by at least one mutation in the mitochondrial sequence with respect to the reference sequence. The definition of new sub-haplogroups must rely on one or more than one mutations that are not considered as hotspots [69]. These caveats prevent phylogenetic ambiguities and errors in the definition of relationships between mitochondrial sequences. The presence of an asterisk (*) in the notation reflects the ‘*paragroup’* condition, that is, those members that do not belong to any of the sub-haplogroups defined within that lineage. These general notation rules have also been followed in the standardization of the human Y-chromosome phylogeny [70].

### 3.2. Methods and Experimental Strategies

The first studies on the human mtDNA variation relied on the use of *Restriction Fragment Length Polymorphisms* (RFLPs). These initial approaches [7,71] demonstrated a correlation between mtDNA restriction patterns and geographical origins of the analyzed individuals.

**Figure 2:**
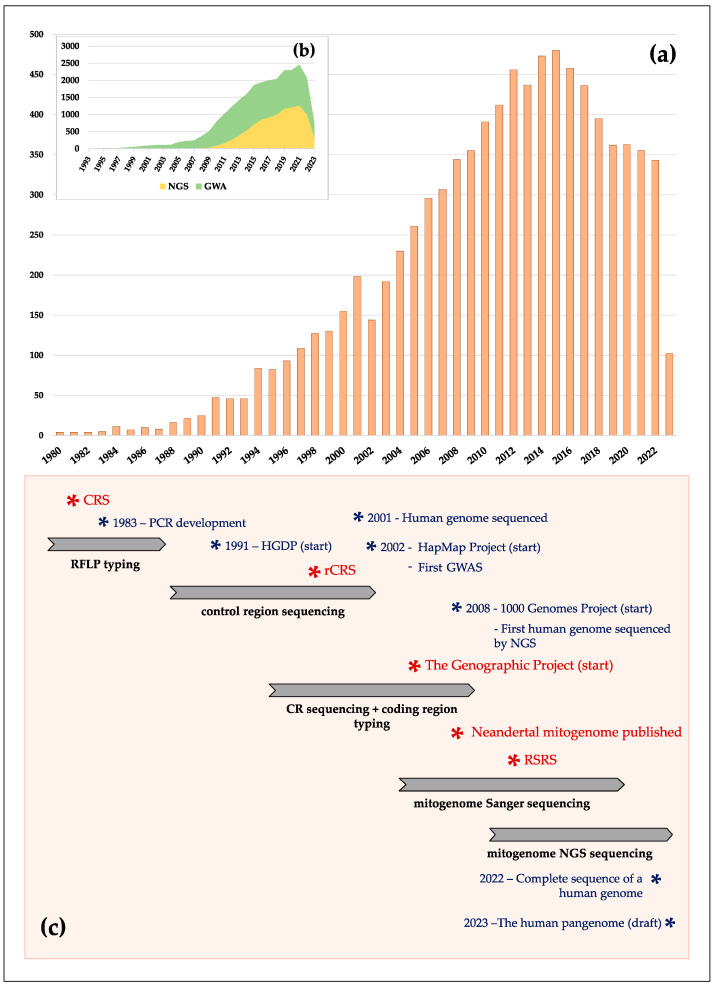
A temporal overview of mtDNA studies in the literature. (**a**) Number of mtDNA studies published per year retrieved from PubMed from 1980 to the present (keywords: “*mitochondrial DNA & human populations*”, accessed on 8 May 2023); (**b**) this panel shows the number of publications, from 1993 to the present, that analyzed human variability with massive genotyping (keywords: “*genome wide analysis and human populations*”) and NGS methods (keywords: “*next-generation sequencing and human populations*”); (**c**) timeline of milestones in mitochondrial studies (in red) with respect to key episodes in human genetics (in blue). Arrows represent different methodologies used for analyzing human mitochondrial diversity as described in the text, and the time span in which they dominate mtDNA research. Abbreviations: GWAS (genome wide association analysis), CR (control region) and HGDP (*Human Genome Diversity Project*).

Lately, attention was paid to the mitochondrial HVR. Its high variability among individuals and populations allowed the detection of specific population patterns and unveiled gene flow scenarios. Control region sequencing was performed in African [72], Asian [73] and European populations [74,75,76,77]. The combination of control region sequences and some variants from the coding region (mostly genotyped by PCR-RFLPs) has been the most usual approach over the 1990s and early 2000s (see Figure 2). This strategy substantially enriched our knowledge of human diversity patterns [78]. 

Multiple genotyping of coding region variants was another later strategy that was especially useful when the control region information itself does not allow a proper discrimination among individuals [79]. Minisequencing methods represent a rapid, robust and cheap way to simultaneously genotype several mtDNA mutations, thus having clear applications in the forensic field [80].

Mitochondrial research over the last 15 years has rest on high-resolution approaches based on complete mitogenomes sequencing. Besides providing a detailed phylogenetic classification as well as the detection of previously overlooked patterns, the study of mitogenomes reveals further insights into the role of natural selection in recent human evolution and the exploration of demographic histories. Usually, these efforts have not been focused on the global mtDNA pool but on the dissection of specific mitochondrial lineages [48,50,66,81,82,83,84,85,86,87,88]. 

Complete mtDNA sequencing was initially developed by the PCR amplification and Sanger sequencing of overlapping fragments. Most studies (from 2006 to 2016, approximately, see Figure 2) followed protocols after Torroni et al. [89], Taylor et al. [90] or Maca-Meyer et al. [91]. The advent of high-throughput (HT) technologies represented a turning point for overcoming time-consuming Sanger mitogenome sequencing. From 2010, the first steps were devoted to heteroplasmy detection, by using Illumina [92] and Roche platforms [93]. These studies demonstrated the presence of higher rates of heteroplasmy with respect to previous works, but also that the sequence errors associated with next-generation sequencing (NGS) methods could be incorrectly read as heteroplasmies. 

From a population viewpoint, one of the first ambitious NGS mitochondrial studies was not haplogroup-based but an analysis of the global mtDNA diversity in a concrete contemporary population [94]. The authors highlighted a convenient switch from the Roche to Illumina platform for gaining coverage and avoiding inconsistencies in *homopolymeric stretches* (the presence of poly-cytosine tracts in certain mitochondrial positions). Likewise, they stressed that the higher error rate of NGS with respect to Sanger sequencing is compensated by the higher coverage per position obtained by using the former methodologies. Therefore, NGS approaches can be considered as more accurate, efficient and rapid than conventional Sanger sequencing.

Paleogenetics also took advantage of the application of new technological breakthroughs to mtDNA analyses. Currently, it is assumed that NGS sequencing is especially suitable for aDNA studies because of its ability to read degraded and short DNA molecules. Some relevant works have explored ancient mitogenomes and benefited from radiocarbon dates obtained from archaeological samples to refine timescales of recent human radiation, as exposed in Section 2.1 [21].

Apart from the Illumina system, Ion Torrent sequencing was also reliable for mitogenome analysis [95]. Moreover, some commercial panels, kits and tools were developed for this purpose, such as the *Precision ID mtDNA Whole Genome Panel* [96,97].

There are two main ways to obtain mitochondrial data from HT methods: to retrieve mtDNA variants from whole genome sequencing (WGS) or to perform a mitochondrial-targeted HT sequencing (as in the cases considered above). Consistent results have been found for haplogroup calling in both methods but with considerable differences with respect to heteroplasmy detection. The higher number of heteroplasmic variants detected with WGS approaches could reveal false positives due to NUMTs or differences in the variant calling algorithms used [98]. Regardless, the extraction of mtDNA reads from WGS reads is now the tendency in human population genetics. 

To a lesser extent, other alternative methods for analyzing mtDNA have also been also. Examples of such technologies include high-performance liquid chromatography (HPLC) [99], high-resolution melt (HRM) profiling [100] or array-based platforms (Affymetrix *MitoChip*, [101,102]).

### 3.3. Databases and Public Repositories

The high amount of data generated in all those studies started to be deposited in specific portals, which have been essential for the mitochondrial scientific community. An up-to-date list of databases and relevant tools is shown in Table 1. 

One of the most frequently used public repository is *PhyloTree*. This site aimed to portray an accurate version of the mtDNA phylogenetic tree with respect to the scientific evidence available so far. The first version was released in August 2008 (*mtDNA tree Build 1*) and contained sequence data from 56 studies. The rCRS/RSRS debate explained in Section 3.1 reached *PhyloTree*, and RSRS was considered the default reference sequence from April 2012 (*mtDNA tree Build 14*), although the site also offered an rCRS-oriented version of the tree. The last release was published in February 2016 (*mtDNA tree Build 17*), comprising 297 references, 24,275 mtDNA sequences and displaying 5500 sub-haplogroups [103]. Each release provided an update of the tree with a growing number of published human mtDNA sequences retrieved from GenBank. Whilst this site is no longer maintained, *PhyloTree* is still a major resource in population genetics, genealogical studies and forensics, since it allows for the standardization of mitochondrial nomenclature, the identification of specific sub-branches and the mtDNA haplogroup assignment. Other tools for lineage classification, such as *HaploGrep* and resources contained in *EMPOP*, are based on *PhyloTree* evidence (see Table 1 for references). The last effort to update and refine the human mtDNA phylogeny was performed in 2021 within the *EMPOP* environment [104].

**Table 1:** Mitochondrial DNA resources and tools (last accessed on 19 May 2023).

Database/Tool	Number of Samples Compiled	Type of Data	Last Update (Version)	Description	Website	Reference	Additional Tools and Resources	Scope
AmtDB (ancient mtDNA database)	2548	Mitogenomes	October 2021	Database of ancient mitochondrial sequences, mostly from Europe.	https://amtdb.org/	[105]	MitoPathoTool (annotation of pathological mtDNA alleles).	Population genetics and mitochondrial disease
EMPOP (EDNAP forensic mtDNA population database)	48,572	Mitogenomes, control region, HVS-I and HVS-II	November 2019 (EMPOP Release 13)	Database of mtDNA sequences.	https://empop.online/	[106]	Haplogroup Browser; EMPcheck (validation of haplotype files); NETWORK (drawing of median networks).	Population genetics and forensics
gnomAD (Genome Aggregation Database)	56,434	Whole genome samples	November 2020 (v3.1 Mitochondrial DNA Variants)	Harmonizing exome and genome sequence data	https://gnomad.broadinstitute.org/downloads#v3-mitochondrial-dna	[107]	-	Nomenclature and notation
HelixMTdb	195,983	14,324 Variants retrieved from mitogenomes	June 2020	Database of mitochondrial DNA variants based on a population of ~195 k individuals.	https://www.helix.com/pages/mitochondrial-variant-database	[108]	-	Population genetics
HmtDB (Human Mitochondrial Genomic Resource)	54,134	Mitogenomes	May 2022	Database of mitochondrial sequences annotated with population and variability data.	https://www.hmtdb.uniba.it/	[109]	Query HmtDB (retrieving data); Genomes classification (haplogroup classification); MToolBox (pipeline to analyze human mtDNA from NGS data); HmtDB Download (download multialignments and site variability data).	Population genetics and mitochondrial disease
HmtVar	54,134	Mitogenomes	October 2022	Explore human mitochondrial variability data and their pathological correlation. Data retrieved from twin database HmtDB	https://www.hmtvar.uniba.it/	[110]	Query HmtVar (retrieving data); HmtVar API (retrieve variants).	Mitochondrial disease
MITOMAP (a human mitochondrial genome database)	59,389	Mostly mitogenomes (>15.4 kbp long)	January 2023	Database of mutations (general variants and pathogenic) in human mitochondrial DNA; reference sequences.	https://www.mitomap.org/MITOMAP	[111]	Allele Search (Search MITOMAP Database for variants at given positions); MITOMASTER (haplogroup classification, variant identification, evaluation of biological significance); Marker Finder (search for variants); Sequence Finder (retrieving Hg-specific GenBank sequences); MitoTIP (pathogenicity scoring of tRNA variants)	Population genetics and mitochondrial disease
MSeqDR: the Mitochondrial Disease Sequence Data Resource Consortium	316,530	Mitogenomes	February 2021	Genome and phenome resource to facilitate clinical diagnosis and research.	https://mseqdr.org/index.php	[112]	Disease Portal (retrieve symptoms, variants from mitochondrial diseases); HPO Browser (human phenotype ontology tree); mvTool (mtDNA variant converter).	Mitochondrial disease
PhyloTree	24,275	Mitogenomes	February 2016 (mtDNA tree Build 17)	Comprehensive and updated of human mitochondrial phylogeny.	https://www.phylotree.org/	[113]	-	Nomenclature and notation
Haplogrep	-	-	April 2023 (Haplogrep 3)	Haplogroup classification service.	https://haplogrep.i-med.ac.at/	[114]	Upload data (FASTA, VCF, txt); haplogroup classification, summary statistics, variant annotations from genome databases; phylogenies.	-
MitoAge	-	-	July 2015 (MitoAge Build 1.0)	Integration of mtDNA sequence data with longevity records.	https://www.mitoage.info/	[115]	Compositional features of mitogenomes, coding and control regions and longevity records for over 900 species (including humans).	-
MitoScape (pipeline for obtaining mtDNA sequences from NGS data)	-	-	March 2021	Software used for obtaining mtDNA sequence data from NGS data.	https://cavatica.sbgenomics.com/public/apps/d3b-bixu/app-publisher/mitoscape-wf	[116]	Alignment, extracting mtDNA sequences, variant calling.	-
Mitoverse	-	-	February 2021	Platform for analyzing mtDNA from NGS and microarray data.	https://mitoverse.i-med.ac.at/index.html#!	[117,118]	Haplocheck (detecting contamination); mtDNA-Server (mtDNA analysis, heteroplasmy identification, contamination).	-

Another searchable, openly accessible database is *MITOMAP*. This site has been regularly updated since 1996. The information provided therein is valuable not only for population geneticists but also for understanding the role of mtDNA in human disease. *MITOMAP* comprises a compendium of tools, datasets and illustrations by which many scholars have learnt mitochondrial genetics.

Interestingly, most of the mitochondrial websites are connected or concurrently use the same resources. The *Haplogrep* tool was designed to assess one key issue in mtDNA studies: the haplogroup classification from sequence information. It was initially released in 2010, as a web application that permitted one to automatically obtain the haplogroup status from the mtDNA variant information uploaded in a formatted text file. It is worth mentioning here that the arrival of NGS methods have transformed the way mtDNA sequences are generated and processed. This fact has permitted developers to their tools accordingly. Hence, from the second release of *Haplogroup* (*Haplogroup* 2), the handling of HT data is supported. 

New resources have arisen to face the challenges of HT data regarding read alignment, heteroplasmy detection and variant annotation. Some pertinent examples are *MToolBox*, *MitoScape* and *Mitoverse* (see Table 1). 

### 3.4. Genetics, Genealogy and Public Engagement 

We cannot neglect here other initiatives where mtDNA have had a leading role. Such projects have represented boundaries between pure scientific research and public outreach—actual ‘*citizen science projects*’. As a paradigm, the *Genographic Project* was initially launched in April 2005 by the National Geographic Society and IBM aiming to build the first ever database of human global genetic patterns and variation. Roughly, 1 million people from more than 140 countries participated in the whole project. The first phase of the project relied on the analysis of both mtDNA (HVR-I) and Y-chromosome markers. The results of this ambitious international project ended up in a plethora of publications. The newly generated data enriched other repositories (*FamilyTree DNA*). Relevant information on mtDNA data can be found in [119]. 

The *Genographic Project* ended and the ancestry kits are no longer available for purchase. Nevertheless, several companies followed in the wake of this approach, leading to an explosion of personal genealogy DNA tests [120], especially in the last decade. Consumers are attracted for several reasons, including the identification of biological relatives, the validation of familiar genealogies or the possible discovery of unexpected ancestry components. These kits are truly valuable for the general society, as they represent vehicles for the transmission of scientific concepts (*ancestry*, *DNA*, *lineage*) and for a realistic understanding of human population history. 

### 3.5. Some Final Thoughts on the Use of mtDNA in Human Phylogeny and Phylogeography

In this section, the particularities that turned mtDNA into a key tool to study genetic relationships within and among human populations have been explored. Nevertheless, there are certain intrinsic limitations and caveats that need to be considered. Firstly, the underlying history of mtDNA as a single locus does not necessarily represent the full picture of a population’s history. Many studies have demonstrated significant differences between maternal (signaled by mtDNA) and paternal (represented by Y-chromosome variation) components. The contrasting patterns should be explained as the result of sex-specific behaviors: different migration rates of females vs. males and differences in effective population sizes [121]. 

Another consideration emerges from the high mitochondrial mutation rate. Recurrent mutations at certain positions of mtDNA sequence (*homoplasy*) can cause phylogenetic ambiguities. This phenomenon leads to situations where several phylogenetic trees could be equally probable when reconstructed from a certain set of mitochondrial sequences [122]. Some of these recurrent mutations [T16519C, T152C, T16311C, T146C, T195C, T16189C (variants scored against the rCRS)] appear more than one hundred times in the human mtDNA phylogeny (see Table 1 in [103]).

## 4. Mitochondrial DNA and Health

The previous sections mainly deal with applications of mtDNA for studying human biological diversity. In addition, the mitochondrial genome is clearly a focus of attention in biomedicine regarding the relevant clinic outcomes of mtDNA mutations and the mitochondrial influence in human aging.

### 4.1. Diseases: Main Features and Population Approaches

In the general population, pathogenic mutations are found in 1/250 individuals, with heteroplasmy levels above 10% [107]. The first diseases linked to mtDNA, Kearns–Sayre syndrome and Leber hereditary optic neuropathy (LHON), were described at the end of 1980 [123,124]. Since then, many primary pathogenic mtDNA mutations—which can compromise OXPHOS and cause disease—have been described [13].

The role of mitochondrial mutations in diseases is complex and mitochondrial disorders have a variable severity and clinical expression. Thus, we can face a broad range of situations, from high penetrance variants to low penetrance risk mutations, which, together with an specific population profile or certain environmental conditions, could lead to disease [125]. 

In this context, there are several features of mtDNA—as a consequence of the organellar location and heteroplasmy—that must be considered: (i) *Threshold effect*: A certain proportion of mutated DNA, with respect to normal mtDNA, is required for the dysfunction to become evident; (ii) *mitochondrial bottleneck*: this phenomenon causes a sampling effect leading to different allele frequencies in the offspring of a maternal lineage; (iii) *mutation accumulation* as a consequence of aging, neurodegeneration and tumorigenesis [27]. 

Mitochondrial disease variants can be point mutations or major rearrangements. Among the latter, the most easily detectable are large-scale partial deletions (Δ-mtDNA), which seem to accumulate throughout individuals’ lifetimes, especially in some tissues. These variants arise as random errors during normal mtDNA replication, and Δ-mtDNA can expand clonally in certain tissues from aged individuals. An open relevant question is focused on whether these variants cause disease or whether they are a consequence of normal aging [13]. 

On the other hand, a variable copy number of mtDNA (mtDNA-CN) has been observed among individuals and cell types. MtDNA-CN is associated with several diseases and phenotypes, and its regulation is influenced by a number of nuclear genes [126,127,128]. 

The role of mtDNA in human disease is highly complex, which can impede proper understanding. Aside from primary mtDNA mutations, there are many mitochondrial variants associated with the predisposition to a broad spectrum of diseases, from neurodegenerative disorders to endocrine or cardiovascular conditions. One very common strategy that actually connects mitochondrial diseases and population approaches is the screening of mtDNA haplogroups in the patients’ sample set in order to check for a possible enrichment (*risk factor*) or decrease (*protective factor*) in lineages when compared to the global mitochondrial pool of a certain ‘*control’* population. Table S1 shows a thorough revision of these studies. Then, mitochondrial haplogroups would act as items representing both views: phylogeographic (since they are linked to a particular territory and different population histories) and functional perspectives (since they are indeed defined by mtDNA mutations that could affect mitochondrial physiology). 

It is worth noting the non-homogeneous results when analyzing the lineage associations within each set of diseases. We can highlight, for instance, the Alzheimer’s disease case, where either risk or beneficial haplogroups across studies are not clear. In a particular case, the association with the lineage U is misleading depending on the sex. In other examples, a certain replicability is observed. For instance, in osteoarthritis, sibling haplogroups J and T are significantly more detected in controls than in patients. In Parkinson’s disease, clade UK appears as a protective factor in some surveys. In general, it is not easy to find solid associations between mtDNA haplogroups and (mainly complex) disorders. 

In a quick inspection to Table S1, lineage J (or the hierarchically upper level, JT) stands out as the most frequent in the ‘*beneficial’* column. Some ‘in vitro’ experiments, for studying the effect of a certain mtDNA in stable nuclear and ambient conditions (*transmitochondrial cybrids*), revealed that clade J cell lines have less OXPHOS capacity and ATP levels than those from haplogroup H, and that such a condition could be relevant in the association with diseases [129]. In this context, haplogroup J would have a pleiotropic effect. 

In order to interpret these results, some key points have been highlighted [129,130,131]. First, the bulk of the considered diseases are complex and multi-factorial. Therefore, several factors, such as other mtDNA or nDNA variants involved; environmental conditions; or the sex and age of individuals, prevent linear relationships between mtDNA composition and these phenotypes. Second, a careful study design is needed with respect to sample sizes, the definition of the experimental groups and statistical tests to be chosen. Case-control strategies are likely affected by population stratification when not properly considering the genomic background of patients. Third, since the mtDNA molecule is totally linked, it is not possible to isolate the effects of single variants from the others that also define a haplogroup. Therefore, clinical mitochondrial research needs to integrate phylogenetic viewpoints since the haplogroup context is relevant to understand the pathogenicity of mtDNA mutations. Finally, the specific lineages detected are directly dependent of the populations screened. In other words, one would not expect to find associations with haplogroups D or F (either positive or negative) in patients with European ancestry, because these lineages are absent in European populations. Consequently, it is problematic to extrapolate findings from one population to another. 

### 4.2. Aging and Longevity

The mitochondrial role in the aging process can be studied by analyzing the changes in the mitochondrial genetic material (see below) or by considering cellular aspects as: (i) *mitochondrial dynamics* (mitochondrial fission, fusion and trafficking are altered with age [132]); (ii) the communication of mitochondria with other organelles (*mito-organellar* processes are key to keep cellular homeostasis and organismal fitness) and (iii) the relationship between mtDNA and nDNA (an impaired *mitonuclear* communication is implicated in aging and age-dependent diseases) [133].

In the classic paper “The Hallmarks of Aging” [134], recently revisited [135], López-Otín et al. underlined the relevance of mtDNA in two of the nine common denominators of aging in mammals: “*genomic instability*” and “*mitochondrial dysfunction*”. The progressive accumulation of somatic mtDNA mutations leads to an inexorable decrease in the mitochondrial function. In this context, *reactive oxygen species* (ROS), generated at low levels because of the mitochondrial respiratory chain (RC) function, can cause somatic mtDNA mutations. These changes in the mtDNA sequence can then provoke RC dysfunction and an increased ROS production, with the progressive accumulation of more mutations, in an unstoppable cascade that results in cellular and tissue failure [28]. However, the basics of MFRTA are being challenged by much evidence. ROS nowadays are not considered as the ultimate responsible factors for mtDNA alterations. In contrast, several studies are demonstrating that probably mtDNA mutations are generated by replication errors rather than by oxidative damage [136]. On the other hand, the effect of mtDNA mutations on ROS production heavily depends on the RC complex affected, whereas random mitochondrial mutations are not associated with increased oxidative stress [137]. Moreover, experimental mammal models demonstrated that antioxidant modifications do not necessarily affect lifespan, and some other reports indicated that the administration of antioxidants might have negative effects [138]. 

Even though the situation is clearly complex, as mtDNA is involved in human aging, one may wonder whether elderly people harbor a specific mitochondrial composition. Many studies focused on centenarians addressed this question. Table 2 shows a clear dependency of findings with respect to the geographic origin of target individuals. Centenarians tend to have an enrichment of haplogroup J—for individuals of European ancestry—or D—for Asian populations, reflecting a population specificity of associations. 

The results observed seem to be related to the phylogenetic definition of these mitochondrial branches. Both lineages are characterized by mutations that affect OXPHOS complex I [haplogroup J: A10398G (*MT-ND3*), A12612G (*MT-ND5*) and G13708A (*MT-ND5*); haplogroup D: C51784a (*MT-ND2*); mutations scored against the rCRS]. Then, a connection is inferred between changes in energy cellular pathways and longevity. However, some authors have also noticed that mitochondrial influence in longevity depends on the interaction between mtDNA variants belonging to different genes. The simultaneous occurrence of mutations on complexes I and III (that, for instance, occur in some internal radiations of haplogroup J, J1c: T14798C (*MT-CYB*) and J2b: G15812A (*MT-CYB*))] are thought to explain some contradictory results. A visible example is the lack of a beneficial effect of haplogroup J in some European populations [149,150]. In accordance with these interpretations, whilst mutations in complex I could be beneficial for longevity, the effect is diluted when combined with mutations in complex III. In addition, it should be important to not only consider interactions between mitochondrial variants, but also epistatic effects of mtDNA-nDNA interactions [149].

Finally, we must consider here—mirroring those exposed in Section 4.1 for complex diseases—that human longevity is a complex and multi-layered phenomenon, where both genetic and environmental factors can severely affect human lifespan. Therefore, it is really challenging to directly model the effects of mtDNA variants for reaching extreme ages.

## 5. Present and Future of Mitochondrial Studies

### 5.1. MtDNA in Current Human Population Genetics

The post-genomic era is driving us to the use of more refined approaches of human genetic diversity, by HT sequencing methods, microarray SNP genotyping and by exploring public datasets of present-day and aDNA human data. Very recently, human population genomic studies have also been immersed in a multi-omics perspective [151,152,153].

The change of paradigm in the field—from uniparental-based studies to high-resolution autosomal genomic analyses—is basically based on the following points: (i) HT technologies are becoming cheaper and more easily accessible for researchers in the last years, (ii) the development of sophisticated bioinformatics tools allow quick and effective analysis of biological big data and (iii) the scientific recognition of the intrinsic limitations of haploid markers, given that they represent sex-biased views of human history. However, it would pertinent to briefly emphasize here the scientific relevance that mitochondrial data can have, even in the present state-of-the-art omics context. 

First, when trying to understand specific sex-dependent behaviors or to develop kinship analyses (see a reflection from the Y chromosome viewpoint in [154]). 

Second, mtDNA allows a direct dating of deep rooting coalescence times and admixture episodes, as this molecule escapes from recombination. The molecular dating from autosomal data based on the analysis of ancestry segments (haplotypes) is limited above a certain number of generations [155], making detecting old events (>4 kya) unreliable. Several efforts are capturing basal events of human evolution by considering both modern and ancient genomes and different methodologies of age estimates [156,157]. However, the use of mitochondrial sequences can overcome the extremely complex and challenging quest of setting deep time boundaries from genomic data. 

Third, mitogenome studies are obviously relevant in human populations from understudied geographic areas and for addressing specific issues regarding the evolutionary past of populations, such as colonization events [158] or adaptation to concrete environments [159]. Some interesting lineages are still being described. A new major branch defined within macro-haplogroup L [160] illustrate that the human mitochondrial phylogeny keeps growing. 

Fourth, mtDNA have a special strength with respect to two specific applications of human genetics. Several strategies are described for performing efficient mtDNA NGS sequencing and standardization data analysis in forensic sciences (see, for instance, [161] and Genes’ Special Issue “Forensic Mitochondrial Genomics”; https://www.mdpi.com/journal/genes/special_issues/forensic_mitochondrial_genomics) (accessed on 13 July 2023) and in the aDNA analysis [162]. 

Finally, it is worth mentioning that mitochondrial evidence drawn over the last decades, in many case studies, are being supported by new data coming from HT methodologies. This is from the big picture (African common genetics roots, see [7,163] and [157,164]) to more specific geographic contexts (the genetic diversity of the Iberian Peninsula, see [88,165] and [166,167]). This last issue justifies that human mitochondrial variation remains valid for approaching the evolutionary history of populations.

### 5.2. The Mitochondrial Genome in Human Health

There are other interesting issues in mitochondrial biology not considered in this review, but that have relevance in the pathogenesis of mitochondrial diseases and are under active investigation. 

For instance, the role of epigenetic modifications in mtDNA disorders is an example, since methylation patterns could explain the phenotypic heterogeneity, variable penetrance and environmental dependence that characterizes this group of diseases [8]. A better understanding of the regulation of mtDNA gene expression can be important in the diagnosis and therapeutic strategies for mitochondrial diseases [168]. 

Other relevant novel perspectives on the role of mtDNA in human health are linked to its relationship with the innate immune response and inflammatory processes [169]. The ectopic presence of mtDNA in the cytosol triggers cellular pathways involved in inflammation, mirroring the situation that occurs when the cell is defending against DNA-containing pathogens [170]. The cGAS-STING signaling pathway (Cyclic GMP–AMP synthase/Stimulator of interferon genes), for instance, represents an axis of regulation of type I IFN (interferon) responses. MtDNA can be released from the organelle into the cytosol or the extracellular space under specific conditions (cellular stress and mitochondrial dysfunction). These biological cascades, although not fully understood, are becoming recognized as targets for therapies for inflammatory and neurodegenerative diseases.

Regarding the clinical interpretation of heteroplasmic mutations in patients by means of HT technologies, some authors claim the necessity of confirmatory studies that take into account the false positives associated with NUMTs [107].

Recently, a NUMTs screening in WGS data, within the frame of the 100,000 Genomes Project, has been published [171]. The authors reported interesting results, such as an overwhelming presence of these fragments across individuals and the detection of very uncommon NUMTs that revealed that the NUMT formation is an “ongoing process”. Therefore, this phenomenon has a clear potential to affect the mitochondrial etiology of age-associated diseases. There is much evidence of the involvement of NUMTs in, for example, human cancer. Processed as the disruption of tumor suppressor genes, the activation of oncogenes or the induction of gene fusion are associated to the presence of these nuclear copies of mtDNA [172]. All these signals justify the need of reaching “*an integrative approach to mitochondrial biology*” [2] that allows merging concepts such pleiotropic effects of mtDNA variants, morphological and functional changes of the organelle, tissue-specific mitochondrial phenotype and mitonuclear epistasis, in order to properly comprehend the role of the so-called “*multifaceted mitochondria*” in human health.

## Figures and Tables

**Table 2:** Relationship between mitochondrial DNA and longevity. Studies were selected considering a sample size > 50. A dash indicates no haplogroup enrichment. The term “y” indicates years old.

Population	N	Enriched Hg	Reference
NE Spain (>85 y)	138 (138 controls)	J (J2)	[139]
C Spain (>100 y)	65 (138 controls)	-	[140]
Italy (>100 y)	212 (275 controls)	J	[141]
Finland (>90 y)	225 (400 controls)	J, UK	[142]
Japan (>100 y)	96	D4a, D4b2b, D5	[143]
Japan (>105 y)	112	D4a	[144]
Japan (>100 y)	96	D4a, D4b2b, D5	[145]
S China (>100 y)	367 (371 controls)	F (females)	[146]
China (>100 y)	402 (458 controls)	-	[147]
Amish (US) (>80 y)	74	X	[148]

## Data Availability

No new data was created in this study.

## Supplementary Materials

The following supporting information can be downloaded at: https://www.mdpi.com/article/10.3390/genes14081534/s1, Table S1: Compendium of different studies that linked human disease with mtDNA haplogroups. References [145,173,174,175,176,177,178,179,180,181,182,183,184,185,186,187,188,189,190,191,192,193,194,195,196,197,198] are cited in the supplementary materials.
