# Supplementary material for: Mitochondrial DNA in Human Diversity and Health: From the Golden Age to the Omics Era
Source: Genes (Basel). 2023 Jul 27;14(8):1534. doi: 10.3390/genes14081534 (PMC10453943; doi:10.3390/genes14081534)
Supplement: Supplementary file 1 [file genes-14-01534-s001.zip › genes-2486314-supplementary.pdf]

Supplementary Material

Mitochondrial DNA in human diversity and health: from the golden age to the omics era

Candela L. Hernández

Table S1. Compendium of different studies that linked human disease with the prevalence of mtDNA haplogroups. The Table is ordered by disorders. Studies were selected considering sample sizes >50. A dash indicates no association.

Population	Disease	N	Risk Hg	Protective Hg	Reference
China (SW, E)	Alzheimer's disease	712 patients / 905 controls	B5	-	[1]
Japan	Alzheimer's disease	96 patients /96 controls	-	-	[2]
Tunisia	Alzheimer's disease	58 patients / 196 controls	-	-	[3]
United States (European ancestry)	Alzheimer's disease	989 patients / 328 controls	U (males)	U (females)	[4]
Finland (Northern Ostrobothnia)	Alzheimer's disease; frontotemporal lobar degeneration (FTLD)	Patients: 128 + 66 / 99 controls	I, W, X (FTLD)	-	[5]
European (undefined)	Breast cancer (BRCA2 mutation carriers)	11,421 patients / 10,793 controls	-	T1a1	[6]
India (N)	Breast cancer	124 patients / 273 controls	N	-	[7]
China (S)	Colorectal, thyroid and breast cancer	Patients: 108 + 100 + 104 / Controls: 124 + 138 + 114	M, D5 (breast), D4a (thyroid)	-	[8]
United States	Cardiovascular diseases	322	-	JT (J)	[9]
Denmark	Risk of ischemic cardiovascular disease (longitudinal study)	9,254 general population	-	-	[10]
Spain (Asturias)	Hypertrophic cardiomyopathy	130 patients / 300 controls	T	-	[11]

Population	Disease	N	Risk Hg	Protective Hg	Reference
Denmark	Hypertrophic cardiomyopathy	91 patients / 9,455 controls	H	J, UK	[12]
India	Hypertrophic cardiomyopathy	114 patients	-	-	[13]
Spain	Ischemic cardiomyopathy	358 patients / 423 controls	H	J	[14]
Austria	Coronary artery disease; diabetic retinopathy	Patients: 487+ 149 / 1,527 controls	T	-	[15]
Spain (NC)	Myocardial infarction, stroke	Patients: 211 + 154 / 154 controls	H (in MI)	J (in hypertension)	[16]
United States	Ewing sarcoma	420 patients	Enriched European Hgs in patients		[17]
Finland, Italy, Norway, Sweden, UK, US, Germany	Multiple sclerosis	7,391 patients / 14,568 controls	JT	-	[18]
Spain, China, US, UK, Netherlands, Korea (meta-analysis)	Osteoarthritis	3,617 patients / 1,572 controls	-	JT (B, G in Asian populations)	[19]
European ancestry	Osteoarthritis	891 patients	-	JT	[20]
Poland	Parkinson's Disease	241 patients / 277 controls		U4, U5a1, K, J1c, J2	[21]
Italy	Parkinson's Disease	620 patients / 1,486 controls	-	K	[22]
Russia (Tatar)	Parkinson's Disease	157 patients / 183 controls	H	UK	[23]
United States (European ancestry)	Parkinson's Disease	609 patients / 340 controls	H	J	[24]
Ashkenazi, Sephardic and North African Jewish	Type 2 diabetes mellitus	1,179	-	N1b1 (in Ashkenazi)	[25]
Japan & Korea	Type 2 diabetes mellitus; atherothrombotic cerebral infarction; metabolic syndrome; myocardial infarction	Patients: 4,271 + 1,081 + 1,337 + 2,137 / 2,250 controls	F (T2DM), D5 (T2DM)	N9a (T2DM, MS in women), N9b (MI)	[26]
Brazil	Type 2 diabetes mellitus	347 patients / 350 controls	JT	-	[27]

References

1. Bi, R.; Zhang, W.; Yu, D.; Li, X.; Wang, H.Z.; Hu, Q.X.; Zhang, C.; Lu, W.; Ni, J.; Fang, Y.; et al. Mitochondrial DNA Haplogroup B5 Confers Genetic Susceptibility to Alzheimer's Disease in Han Chinese. *Neurobiol. Aging* **2015**, *36*, 1604.e7-1604.e16, doi:10.1016/j.neurobiolaging.2014.10.009.

2. Wong, J.; Id, J.S.S.; Pienaar, I.S.; Id, J.L.E. Mitochondrial DNA Population Variation Is Not Associated with Alzheimer's in the Japanese Population : A Consistent Finding across Global Populations. *PLoS One* **2022**, 1–9, doi:10.1371/journal.pone.0276169.

3. Salem, N. Ben; Boussetta, S.; Rojas, I. De; Grau, S.M.; Montrreal, L.; Mokni, N.; Mahmoud, I.; Younes, S.; Daouassi, N.; Frih, M.; et al. Mitochondrial DNA and Alzheimer's Disease : A First Case – Control Study of the Tunisian Population. *Mol. Biol. Rep.* **2022**, *49*, 1687–1700, doi:10.1007/s11033-021-06978-7.

4. van der Walt, J.M.; Dementieva, Y.A.; Martin, E.R.; Scott, W.K.; Nicodemus, K.K.; Kroner, C.C.; Welsh-Bohmer, K.A.; Saunders, A.M.; Roses, A.D.; Small, G.W.; et al. Analysis of European Mitochondrial Haplogroups with Alzheimer Disease Risk. *Neurosci. Lett.* **2004**, *365*, 28–32, doi:10.1016/j.neulet.2004.04.051.

5. Krüger, J.; Hinttala, R.; Majamaa, K.; Remes, A.M. Mitochondrial DNA Haplogroups in Early-Onset Alzheimer's Disease and Frontotemporal Lobar Degeneration. *Mol. Neurodegener.* **2010**, *5*, 1–6, doi:10.1186/1750-1326-5-8.

6. Blein, S.; Bardel, C.; Danjean, V.; McGuffog, L.; Healey, S.; Barrowdale, D.; Lee, A.; Dennis, J.; Kuchenbaecker, K.B.; Soucy, P.; et al. An Original Phylogenetic Approach Identified Mitochondrial Haplogroup T1a1 as Inversely Associated with Breast Cancer Risk in BRCA2 Mutation Carriers. *Breast Cancer Res.* **2015**, *17*, 1–15, doi:10.1186/s13058-015-0567-2.

7. Darvishi, K.; Sharma, S.; Bhat, A.K.; Rai, E.; Bamezai, R.N.K. Mitochondrial DNA G10398A Polymorphism Imparts Maternal Haplogroup N a Risk for Breast and Esophageal Cancer. *Cancer Lett.* **2007**, *249*, 249–255, doi:10.1016/j.canlet.2006.09.005.

8. Fang, H.; Shen, L.; Chen, T.; He, J.; Ding, Z.; Wei, J.; Qu, J.; Chen, G.; Lu, J.; Bai, Y. Cancer Type-Specific Modulation of Mitochondrial Haplogroups in Breast, Colorectal and Thyroid Cancer. *BMC Cancer* **2010**, *10*, 421, doi:10.1186/1471-2407-10-421.

9. Veronese, N.; Stubbs, B.; Koyanagi, A.; Vaona, A.; Demurtas, J.; Schofield, P.; Maggi, S. Mitochondrial Genetic Haplogroups and Cardiovascular Diseases: Data from the Osteoarthritis Initiative. *PLoS One* **2019**, *14*, 4–13, doi:10.1371/journal.pone.0213656.

10. Benn, M.; Schwartz, M.; Nordestgaard, B.G.; Tybjærg-Hansen, A. Mitochondrial Haplogroups: Ischemic Cardiovascular Disease, Other Diseases, Mortality, and Longevity in the General Population. *Circulation* **2008**, *117*, 2492–2501, doi:10.1161/CIRCULATIONAHA.107.756809.

11. Castro, M.G.; Huerta, C.; Reguero, J.R.; Soto, M.I.; Doménech, E.; Álvarez, V.; Gómez-Zaera, M.; Nunes, V.; González, P.; Corao, A.; et al. Mitochondrial DNA Haplogroups in Spanish Patients with Hypertrophic Cardiomyopathy. *Int. J. Cardiol.* **2006**, *112*, 202–206, doi:10.1016/j.ijcard.2005.09.008.

12. Hagen, C.M.; Aidt, F.H.; Hedley, P.L.; Jensen, M.K.; Havndrup, O.; Kanters, J.K.; Moolman-Smook, J.C.; Larsen, S.O.; Bundgaard, H.; Christiansen, M. Mitochondrial Haplogroups Modify the Risk of Developing Hypertrophic Cardiomyopathy in a Danish

Population. *PLoS One* **2013**, *8*, e71904, doi:10.1371/journal.pone.0071904.

13. Govindaraj, P.; Khan, N.A.; Rani, B.; Rani, D.S.; Selvaraj, P.; Jyothi, V.; Bahl, A.; Narasimhan, C.; Rakshak, D.; Premkumar, K.; et al. Mitochondrial DNA Variations Associated with Hypertrophic Cardiomyopathy. *Mitochondrion* **2014**, *16*, 65–72, doi:10.1016/j.mito.2013.10.006.

14. Fernández-Caggiano, M.; Barallobre-Barreiro, J.; Rego-Pérez, I.; Crespo-Leiro, M.G.; Paniagua, M.J.; Grillé, Z.; Blanco, F.J.; Doménech, N. Mitochondrial Haplogroups H and J: Risk and Protective Factors for Ischemic Cardiomyopathy. *PLoS One* **2012**, *7*, 1–7, doi:10.1371/journal.pone.0044128.

15. Kofler, B.; Mueller, E.E.; Eder, W.; Stanger, O.; Maier, R.; Weger, M.; Haas, A.; Winker, R.; Schmut, O.; Paulweber, B.; et al. Mitochondrial DNA Haplogroup T Is Associated with Coronary Artery Disease and Diabetic Retinopathy: A Case Control Study. *BMC Med. Genet.* **2009**, *10*, 1–7, doi:10.1186/1471-2350-10-35.

16. Umbria, M.; Ramos, A.; Caner, J.; Vega, T.; Lozano, J.E.; Santos, C.; Aluja, M.P. Involvement of Mitochondrial Haplogroups in Myocardial Infarction and Stroke: A Case-Control Study in Castile and Leon (Spain) Population. *Mitochondrion* **2019**, *44*, 1–6, doi:10.1016/j.mito.2017.12.004.

17. Kaneva, K.; Schurr, T.G.; Tatarinova, T. V; Buckley, J.; Merkurjev, D.; Triska, P.; Liu, X.; Done, J.; Maglinte, D.T.; Deapen, D.; et al. Mitochondrial DNA Haplogroup, Genetic Ancestry, and Susceptibility to Ewing Sarcoma. *Mitochondrion* **2022**, *67*, 6–14, doi:10.1016/j.mito.2022.09.002.

18. Tranah, G.J.; Santaniello, A.; Caillier, S.J.; D'Alfonso, S.; Boneschi, F.M.; Hauser, S.L.; Oksenberg, J.R. Mitochondrial DNA Sequence Variation in Multiple Sclerosis. *Neurology* **2015**, *85*, 325–330, doi:10.1212/WNL.0000000000001744.

19. Zhao, Z.; Li, Y.; Wang, M.; Jin, Y.; Liao, W.; Zhao, Z.; Fang, J. Mitochondrial DNA Haplogroups Participate in Osteoarthritis: Current Evidence Based on a Meta-Analysis. *Clin. Rheumatol.* **2020**, *39*, 1027–1037, doi:10.1007/s10067-019-04890-x.

20. Soto-Hermida, A.; Fernández-Moreno, M.; Oreiro, N.; Fernández-López, C.; Pértega, S.; Cortés-Pereira, E.; Rego-Pérez, I.; Blanco, F.J. Mitochondrial DNA (MtDNA) Haplogroups Influence the Progression of Knee Osteoarthritis.Data from the Osteoarthritis Initiative (OAI). *PLoS One* **2014**, *9*, e112735, doi:10.1371/journal.pone.0112735.

21. Gaweda-Walerych, K.; Maruszak, A.; Safranow, K.; Bialecka, M.; Klodowska-Duda, G.; Czyzewski, K.; Slawek, J.; Rudzinska, M.; Styczynska, M.; Opala, G.; et al. Mitochondrial DNA Haplogroups and Subhaplogroups Are Associated with Parkinson's Disease Risk in a Polish PD Cohort. *J. Neural Transm.* **2008**, *115*, 1521–1526, doi:10.1007/s00702-008-0121-9.

22. Ghezzi, D.; Marelli, C.; Achilli, A.; Goldwurm, S.; Pezzoli, G.; Barone, P.; Pellecchia, M.T.; Stanzione, P.; Brusa, L.; Bentivoglio, A.R.; et al. Mitochondrial DNA Haplogroup K Is Associated with a Lower Risk of Parkinson's Disease in Italians. *Eur. J. Hum. Genet.* **2005**, *13*, 748–752, doi:10.1038/sj.ejhg.5201425.

23. Khusnutdinova, E.; Gilyazova, I.; Ruiz-Pesini, E.; Derbeneva, O.; Khusainova, R.; Khidiyatova, I.; Magzhanov, R.; Wallace, D.C. A Mitochondrial Etiology of Neurodegenerative Diseases: Evidence from Parkinson's Disease. *Ann. N. Y. Acad. Sci.* **2008**, *1147*, 1–20, doi:10.1196/annals.1427.001.

24. van der Walt, J.M.; Nicodemus, K.K.; Martin, E.R.; Scott, W.K.; Nance, M.A.; Watts, R.L.;

Hubble, J.P.; Haines, J.L.; Koller, W.C.; Lyons, K.; et al. Mitochondrial Polymorphisms Significantly Reduce the Risk of Parkinson Disease. *Am. J. Hum. Genet.* **2003**, *72*, 804–811.

25. Feder, J.; Blech, I.; Ovadia, O.; Amar, S.; Wainstein, J.; Raz, I.; Dadon, S.; Arking, D.E.; Glaser, B.; Mishmar, D. Differences in MtDNA Haplogroup Distribution among 3 Jewish Populations Alter Susceptibility to T2DM Complications. *BMC Genomics* **2008**, *9*, 198, doi:10.1186/1471-2164-9-198.

26. Nishigaki, Y.; Fuku, N.; Tanaka, M. Mitochondrial Haplogroups Associated with Lifestyle-Related Diseases and Longevity in the Japanese Population. *Geriatr. Gerontol. Int.* **2010**, *10*, S221–S235, doi:10.1111/j.1447-0594.2010.00599.x.

27. Crispim, D.; Canani, L.H.; Gross, J.L.; Tschiedel, B.; Souto, K.E.P.; Roisenberg, I. The European-Specific Mitochondrial Cluster J/T Could Confer an Increased Risk of Insulin-Resistance and Type 2 Diabetes: An Analysis of the m.4216T > C and m.4917A > G Variants. *Ann. Hum. Genet.* **2006**, *70*, 488–495, doi:10.1111/j.1469-1809.2005.00249.x.
